# Signal and noise extraction from analog memory elements for neuromorphic computing

**DOI:** 10.1038/s41467-018-04485-1

**Published:** 2018-05-29

**Authors:** N. Gong, T. Idé, S. Kim, I. Boybat, A. Sebastian, V. Narayanan, T. Ando

**Affiliations:** 1 IBM T. J. Watson Research Center, 1101 Kitchawan Road, Yorktown Heights, NY 10598 USA; 2 Department of Electrical Engineering, Yale University, 10 Hillhouse Avenue, New Haven, CT 06511 USA; 3 IBM Research-Zurich, Säumerstrasse 4, 8803 Rüschlikon, Switzerland; 4 Ecole Polytechnique Federale de Lausanne (EPFL), Route Cantonale, 1015 Lausanne, Switzerland

## Abstract

Dense crossbar arrays of non-volatile memory (NVM) can potentially enable massively parallel and highly energy-efficient neuromorphic computing systems. The key requirements for the NVM elements are continuous (analog-like) conductance tuning capability and switching symmetry with acceptable noise levels. However, most NVM devices show non-linear and asymmetric switching behaviors. Such non-linear behaviors render separation of signal and noise extremely difficult with conventional characterization techniques. In this study, we establish a practical methodology based on Gaussian process regression to address this issue. The methodology is agnostic to switching mechanisms and applicable to various NVM devices. We show tradeoff between switching symmetry and signal-to-noise ratio for HfO_2_-based resistive random access memory. Then, we characterize 1000 phase-change memory devices based on Ge_2_Sb_2_Te_5_ and separate total variability into device-to-device variability and inherent randomness from individual devices. These results highlight the usefulness of our methodology to realize ideal NVM devices for neuromorphic computing.

## Abstract (web summary)

The application of resistive and phase-change memories in neuromorphic computation will require efficient methods to quantify device-to-device and switching variability. Here, the authors assess the impact of a broad range of device switching mechanisms using machine learning regression techniques.

## Introduction

**Fig. 1:**
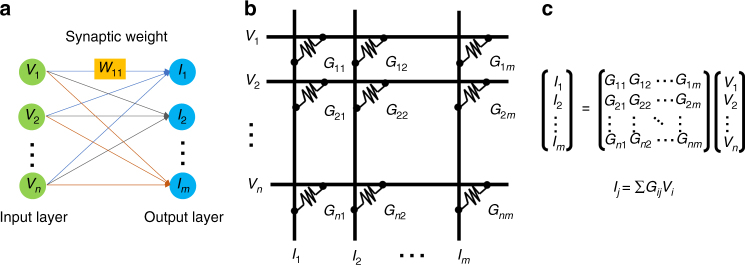
Neuromorphic computing system based on NVM. **a** Schematic illustration of one-layer neural network with synaptic weights (*W*) connecting an input layer to an output layer. **b** A synaptic weight is represented by a conductance value of an NVM element at each cross-point in a crossbar array structure. **c** Vector-matrix multiplication is performed by sensing the current (*I*) for each column, which is the product of the synaptic weight (*G*) and the input signal (*V*)

Over several decades, the von Neumann architecture has enabled exponential improvements in system performance. However, as device scaling has slowed and demand to handle big data has soared, the time and energy spent transporting data across the physically separated memory and processing units have started to limit the performance and power efficiency. As potential alternatives, neuro-inspired non-von Neumann computing paradigms have become promising candidates to perform real-world tasks^1, 2^. One avenue of research is referred to as in-memory computing or computational memory, which exploits the physical properties of non-volatile memory (NVM) devices for both storing and processing information^3–6^. Recently, a large-scale experimental demonstration of this concept using an array of one million phase-change memory (PCM) devices has been reported^7^. Another paradigm is hardware acceleration of deep neural network (DNN)^8–12^ training via the use of dense crossbar arrays of NVM to perform locally analog computation at the location of the data. As shown in Fig. 1, it is possible to use NVM devices with variable conductance states, such as resistive random access memory (ReRAM)^13^ and PCM^14^ to represent the synaptic weights and to perform vector-matrix multiplication using the basic electrical principles, i.e., Ohm’s and Kirchhoff’s laws, thus enabling local and parallel computation on a large scale. By making the conductance change of the NVM element bidirectional, backpropagation algorithm can be implemented. Such a crossbar array of NVMs is expected to achieve significant acceleration factors of DNN training and remarkable reduction in power and area^15, 16^. Another active area of research is spiking neural networks (SNNs) motivated by the need to build more biologically realistic neural network models. Several neuromorphic computing platforms are being developed which are optimized for emulating spike-based computation. These SNNs are typically trained using certain local update rules, such as the spike-timing-dependent plasticity. NVM devices have recently found applications as both synaptic and neuronal elements of such SNNs^17–20^.

The key technical challenge for these applications is to realize ideal NVM elements with continuous (analog-like) conductance tuning capability in response to electrical pulses with acceptable noise levels. For acceleration of DNN training, symmetric conductance change with positive and negative pulse amplitudes is another key requirement^15, 16^. The device conductance should go up with a voltage pulse of one polarity and should go down by the same magnitude with a voltage pulse of the opposite polarity. In general, NVM elements do not show this symmetric switching behavior. Therefore, a differential approach is often used in which two conductance values are compared in a unit cell^14^. In this configuration, linearity in switching is required to ensure a symmetric differential signal. In reality, most NVM elements exhibit highly non-linear evolution of conductance as a function of the number of consecutively applied pulses. This results in significant errors in weight updates^13^. In addition, such non-linear conductance change makes separation of signal and noise extremely difficult. Most NVM elements show stochasticity related to the physical origins of switching. When incremental weight updates are performed for analog NVM devices, the magnitude of conductance change approaches the level of inherent randomness^21^, manifesting as significant noise components. Therefore, establishing a universally applicable methodology to evaluate signal-to-noise ratio (SNR) of non-linear and analog NVM devices is of paramount importance for neuromorphic computing applications.

In this study, we first establish a practical methodology based on a machine learning algorithm to precisely separate signal and noise components from an analog NVM device with non-linear conductance changes. The methodology is agnostic to the device physics, enabling us to apply it to different types of NVM elements. First, the methodology is applied to HfO_2_-based ReRAM to understand the relationship between switching symmetry and SNR. Next, the methodology is applied to PCM devices based on doped-Ge_2_Sb_2_Te_5_ (GST). We characterize 1000 devices and separate device-to-device variability and inherent randomness from individual devices.

## Results

### Analog switching behaviors of ReRAM and PCM

**Fig. 2:**
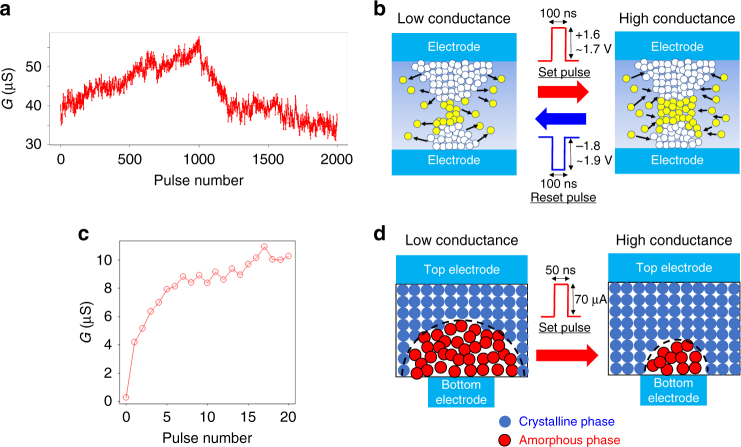
Analog switching behaviors of ReRAM and PCM. **a** Device *G* as a function of pulse number for our HfO_2_-based ReRAM device. 1000 consecutive set pulses, followed by 1000 consecutive reset pulses were applied on the top electrode. **b** The change of *G* is attributed to change in the configuration of the current conducting filament which consists of oxygen vacancies as schematically illustrated. **c** Device conductance *G* as a function of pulse number for our GST-based PCM device. A total of 20 consecutive set pulses were applied. **d** The *G* increase in response to set pulses corresponds to the transition from the amorphous phase to the crystalline phase of GST as schematically illustrated

As shown in Fig. 2a, our ReRAM device exhibited analog-like (incremental) change in the device conductance (*G*) in response to voltage pulses. Consecutive positive voltage (set) pulses (pulse number 1–1000) on the top electrode caused an overall ascending trend of *G* with some pulse-to-pulse fluctuations. On the other hand, consecutive negative voltage (reset) pulses (pulse number 1001–2000) caused a descending trend of *G* with similar fluctuations. The change of *G* in oxide ReRAM device is attributed to change in the configuration of the current conducting filament which consists of oxygen vacancies in a metal oxide film^22, 23^ as schematically illustrated in Fig. 2b. The movement of the oxygen vacancies in response to electrical signals has a probabilistic nature and it emerges as inherent randomness in weight updates, which are superimposed on the expected signal^13^.

As for PCM, we investigated the device *G* changes in response to 20 consecutive set pulses. Figure 2c is a plot of *G* as a function of pulse number, showing incremental changes with a non-linear trace, which is convoluted with pulse-to-pulse fluctuations. The PCM device includes a small part of phase-change material that is sandwiched by top and bottom electrodes. Transition from the low conductance state (amorphous phase) to the high conductance state (crystalline phase) is caused by set pulses that create sufficient joule heating for crystallization of the GST material while the temperature is kept below the melting point as schematically illustrated in Fig. 2d. Due to the stochastic nature in crystallization of the phase-change materials^2, 20, 21, 24, 25^, there is significant randomness associated with the weight updates. On the other hand, reset to the low conductance state requires melting of the GST material and this process is known to be abrupt. For the purpose of characterization of analog switching behaviors, we focused on incremental set operations for PCM in this study.

### Characterization of NVM elements

To evaluate the performance of analog NVM elements for neuromorphic computing applications, one has to extract noise-free signals from experimental data. A conventional approach is to assume a parametric model for expected conductance changes, derived from relatively simple assumptions on underlying physics. For ReRAM devices, an exponential formula has been proposed to capture the non-linear trend^13^. However, the pre-assumed exponential relationship often causes significant errors when fitting weight update as a function of number of applied pulses. In addition, different NVM elements generally need different fitting formulas, making it difficult to compare key performance parameters, such as switching symmetry and SNR, on a common ground. To address this issue, we leverage a machine learning algorithm called Gaussian process regression (GPR)^26^. GPR is a non-parametric Bayesian regression method, which does not assume any specific functional form such as linear and exponential. The main motivation for implementing GPR in the analysis of analog NVM elements is to let experimental data give predictions of noise-free signals by themselves. The major assumption we used is the smoothness of the curve. For analog NVM devices, we exploit continuous changes in switching media (e.g., filament configuration for ReRAM, volume of crystalline region for PCM) rather than non-continuous phenomena to achieve incremental conductance changes. This makes analog switching data highly compatible with the assumption of smoothness. The key ingredient of GPR is the kernel matrix (Eq. (6) in Methods), which controls the smoothness of the estimated functional curve. We established a practical approach to optimize the kernel matrix by combining the Bayesian marginalized likelihood maximization with the frequentists’ cross-validation approach. This enabled us to precisely separate signal and noise for our large dataset while avoiding numerical instability. The proposed inference procedure also assumes that a prior probability distribution over underlying functions follows a multivariate Gaussian distribution, which consists of a linear combination of finite random variables. This assumption is consistent with the switching mechanism of analog memory devices where the device conductance is governed by parallel configurations of randomly distributed conducting filaments comprising oxygen vacancies or crystalline phase-change materials. The measured device conductance values indeed follow a Gaussian distribution around noise-free signals and this was verified by observing the distribution of noise in our experimental data for ReRAM (Supplementary Note 1). The details of our GPR-based methodology are described in Methods section.

**Fig. 3:**
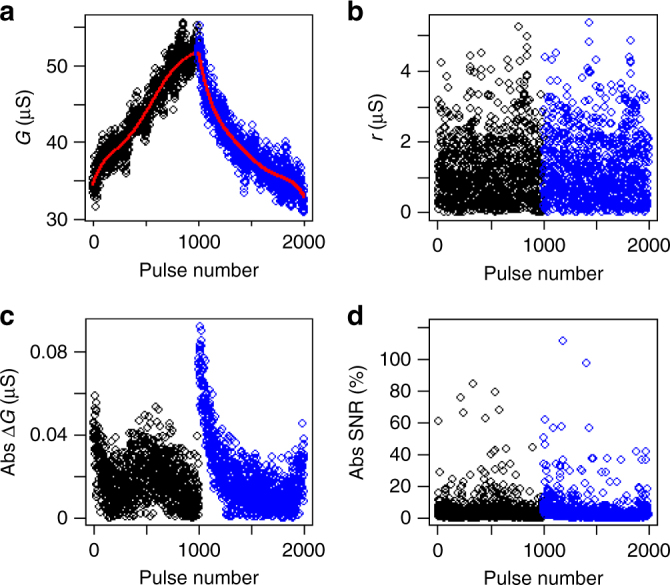
Extraction of key metrics for neuromorphic computing. The GPR-based methodology is applied to our ReRAM data with 1000 consecutive set pulses, followed by 1000 consecutive reset pulses. **a** Device *G* as a function of pulse number for set (black) and reset (blue) pulse sequences. The predicted noise-free signals are shown in red lines. **b** Extracted *r* values as a function of pulse number for set (black) and reset (blue) pulse sequences. **c** Extracted ∆*G* values as a function of pulse number for set (black) and reset (blue) pulse sequences. **d** Extracted SNR values as a function of pulse number for set (black) and reset (blue) pulse sequences

We performed cross-validation^27^ using our ReRAM data and confirmed that the GPR-based methodology extracted the inherent features irrespective of the sampling size (Supplementary Note 2). We confirmed the robustness of our methodology against the variation of duration of input pulses from 5 to 100 ns, covering the range of interest for neuromorphic computing (Supplementary Note 3). We also confirmed the robustness of our methodology against the variation of test temperature (Supplementary Note 4). For the rest of the analysis, we used a pulse duration of 100 ns and tested the devices at room temperature. Next, we extracted key performance metrics using the GPR fitting. We applied the methodology to our ReRAM data with 1000 consecutive set pulses, followed by 1000 consecutive reset pulses, for the purpose of characterizing switching symmetry. As shown in Fig. 3a, the GPR fitting gave predicted noise-free curves (red lines) for both set (black) and reset (blue) pulse sequences. Once the noise-free curves are estimated, the *G* change per pulse, denoted by Δ*G*, is easily computed, based on which we define SNR as

1 $${\mathrm{SNR}}\,\underline{\underline {{\mathrm{def}}}} \,\frac{{{\mathrm{\Delta }}G}}{r},$$

where *r* represents the absolute difference between predicted and observed *G* values (i.e., residuals). The impact of SNR on the accuracy of neural network was previously discussed^21^. Since relatively long sequences were used for both ReRAM and PCM devices to minimize fluctuations in read signals, we attribute *r* to inherent randomness associated with the physical origin of weight update. In artificial neural network implementations, fast reading is particularly preferred to decrease the overall cycle time and consequently accelerate the computational operations. This should increase the contribution of read noise. In this case, we need to optimize the read operation to balance the overall performance and the noise level, which is beyond the scope of this work. The extracted *r* value is shown as a function of pulse number in Fig. 3b. The absolute Δ*G* values for set and reset pulses are denoted by Δ*G*_+_ and Δ*G*_−_, respectively. The Δ*G*_+_ (black) and Δ*G*_−_ (blue) are plotted as a function of pulse number in Fig. 3c. Figure 3d shows absolute SNR, calculated locally at each pulse from Δ*G* and *r*. For characterization of switching symmetry, we introduce symmetry factor (SF), which is defined as

2 $${\mathrm{SF}}\underline{\underline {\,{\mathrm{def}}\,}} \frac{{\Delta G_ + - \Delta G_ - }}{{\Delta G_ + + \Delta G_ - }}.$$

With this definition, the degree of symmetry is quantified as a value between −1 and 1, with 0 corresponding to the perfect symmetry. Asymmetry in both directions (larger Δ*G*_+_ versus Δ*G*_−_) are equally weighted around 0 and can be compared with absolute values. In order to compute SF and SNR at a given *G* level, we need to express Δ*G*_+_, Δ*G*_−_, and *r* as functions of *G*. Therefore, we divided the total *G* range into 100 sub-ranges and computed a mean value of Δ*G* and a root mean square value of *r* within each *G* sub-range. In this way, one can obtain SF and SNR for each *G* sub-range. The local extraction (i.e., at a certain pulse number or *G* level) of SF and SNR is a powerful feature of our methodology. The symmetry requirement for acceleration of DNN training specified in ref. ^15^ (<5% difference between Δ*G*_+_ and Δ*G*_−_) corresponds to |SF| <0.025.

### Switching symmetry and SNR of ReRAM devices

**Fig. 4:**
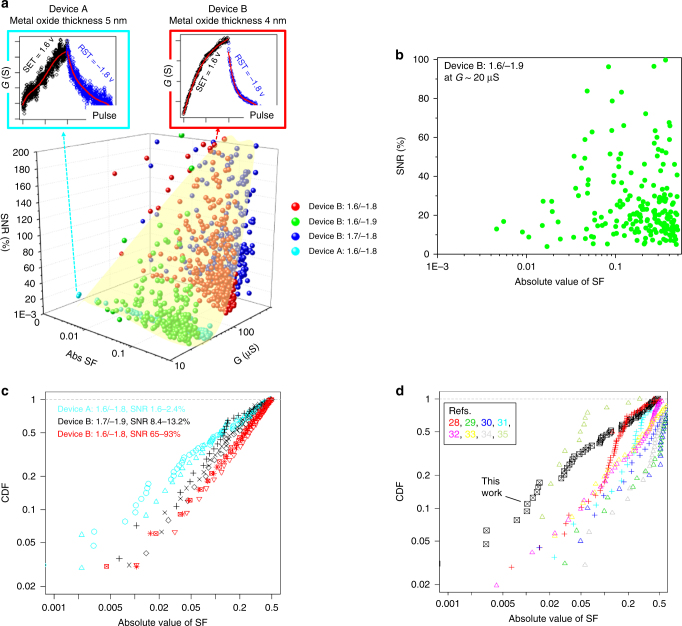
Tradeoff between switching symmetry and SNR for ReRAM. The GPR-based methodology was applied to our ReRAM data with different metal oxide thicknesses (device A: 5 nm, device B: 4 nm) and different set (1.6–1.7 V) and reset (−1.8 to −1.9 V) voltages to investigate the relationships between the key metrics. **a** SNR and SF as functions of *G*. The original data for two representative device and pulse conditions are shown in the insets to contrast the tradeoff. **b** Cross-sectional 2D plot of SNR versus SF taken at *G* ~20 μs from **a**. **c** Plot of cumulative distribution function (CDF) of absolute values of SF for data in different SNR ranges. **d** Benchmark plot of SF. The optimized ReRAM data (device A, set: 1.6 V, reset: −1.8 V) were compared with refs. ^28–35^. The benchmark data points are color-coded (see the legend) and shown as symbols for same amplitude pulses and as crosses for varying amplitude pulses

We applied the GPR-based methodology on our ReRAM devices with different metal oxide thicknesses (device A: 5 nm, device B: 4 nm). The devices were tested under different set and reset voltages and the SNR and SF values were extracted locally at each *G* level, as shown in Fig. 4a. For SNR, we took mean values for set and reset traces. Representative *G* versus pulse number traces are shown in insets. Figure 4b shows a cross-sectional two-dimensional plot of SNR versus SF taken at *G* ~20 μs from Fig. 4a. At this *G* level, low |SF| values were achieved at relatively low SNR values, and vice versa. Data points are absent in the upper-left corner of Fig. 4b, indicating that there is a fundamental tradeoff between SNR and SF values. In order to investigate the relationship between SNR and SF values for multiple device/pulse conditions spanning different *G* levels, they were grouped according to SNR values and cumulative distribution function of |SF| were compared, as shown in Fig. 4c. The reproducibility of the trend was confirmed up to 10 different devices of device type B (Supplementary Note 5). One can clearly observe that the device/pulse conditions that lead to higher SNR values tend to result in poor switching symmetry. The tradeoff can be directly observed in the *G* versus pulse number plots (the insets of Fig. 4a). We speculate that higher switching symmetry is achieved by making the movement of oxygen vacancies more incremental and thereby changing the width of current conducting filament rather than completely rupturing and reforming it. Δ*G* is smaller for the former case and it should eventually approach the level of inherent randomness, resulting in lower SNR values. Such a tradeoff makes it difficult to improve both switching symmetry and SNR at the same time and it remains as a key challenge for ReRAM devices for neuromorphic computing applications. However, if these key metrics are accurately quantified like we demonstrated with our GPR-based methodology, one can optimize the device and pulse conditions to find the optimum point within the tradeoff. As reviewed in a previous section, switching symmetry is a critical requirement to implement backpropagation algorithm for DNNs. In reality, learning accuracy is compromised due to non-ideal (asymmetric) switching characteristics of synaptic elements. Therefore, we optimized the device condition (device A) and the pulse condition (set: 1.6 V, reset: –1.8 V) using the GPR-based methodology to minimize SF. The beauty of our methodology is the capability to extract SF, agnostic to switching mechanisms and irrespective of data size. This enabled us to compare our ReRAM data with various resistive switching devices in literature^28–35^. There have been reports on improved switching symmetry using pulses with varying amplitude^28, 30, 31^. These cases were benchmarked together and marked separately in Fig. 4d. One can see a general trend of improved symmetry using pulses with varying amplitudes. This approach, however, requires sensing of current states of individual devices and adjustment of voltage amplitudes, which is not compatible with local and parallel computation. It should be noted that our optimized ReRAM data showed good switching symmetry compared with all benchmark data with identical voltage pulses. This is a significant step forward to realize online training capability in a parallel manner. Future work needs to focus on simultaneously achieving sufficiently high SNR values with materials optimizations.

### Breakdown of variability components in 90-nm PCM devices

**Fig. 5:**
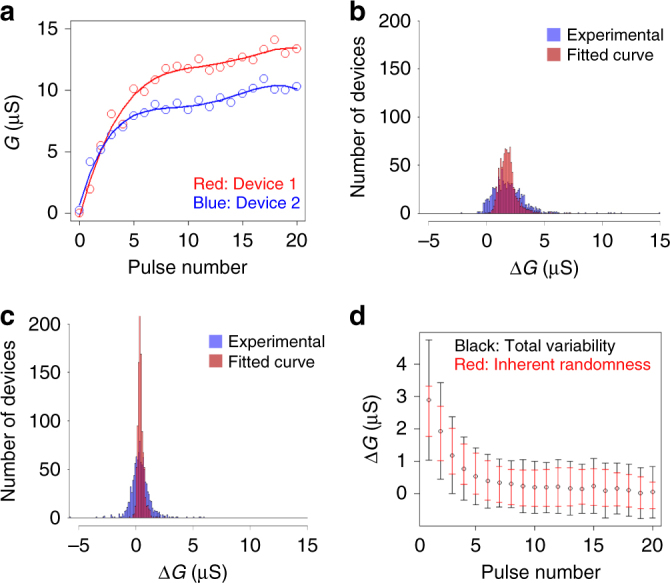
Separation of device-to-device variability and inherent randomness for PCM. **a** Device *G* as a function of pulse number for two representative PCM devices (devices 1 and 2), fabricated with the identical process. The predicted noise-free signals from the GPR fitting are shown in red and blue solid lines. The difference of two fitted lines corresponds to device-to-device variability, whereas the fluctuations of the experimental data around the fitted lines are attributed to inherent randomness from individual devices. Histograms of Δ*G* values extracted from experimental data (blue) and fitted curves (red) (**b**) after the second pulse and (**c**) after the sixth pulse. **d** The mean and standard deviation of Δ*G* obtained from the experimental data (shown in black circles and error bars) were compared with the root mean square of inherent randomness (*r*) obtained from the GPR-based methodology (shown in red error bars) as a function of pulse number. The inherent randomness accounts for 67% of the total variability

A conventional approach to extract inherent randomness associated with weight updates is to test multiple devices and to obtain statistical distributions^21^. The variability obtained in this manner, however, includes device-to-device variability in addition to inherent randomness from individual devices. These variability components need to be quantified separately in order to accurately assess potentials of certain NVM elements for neuromorphic computing applications. We tested 1000 PCM devices and extracted signal and noise from individual devices using our GPR-based methodology. This enabled us to further break down the total variability to the inherent randomness of individual devices and the device-to-device variability. These two variability components are illustrated in Fig. 5a with two representative PCM devices (devices 1 and 2) that were fabricated with the identical process. The GPR fitting was performed to predict noise-free signals as shown in red and blue solid lines, respectively, in Fig. 5a. The predicted signals for devices 1 and 2 deviate from each other due to device-to-device variability. In addition, the experimental data points (shown in circles) fluctuate around the individual fitted lines, which is attributable to inherent randomness of weight updates since the read noise was minimized by the test sequence as described in Methods section. We compared the histograms of Δ*G* values extracted from experimental data and fitted curves after the pulse numbers 2 (Fig. 5b) and 6 (Fig. 5c). The statistical distribution of the fitted curves (red) is the contribution from device-to-device variability, whereas the statistical distribution of the experimental data (blue) includes inherent randomness superimposed on top of that. The latter distribution was much wider, clearly showing significant contribution of inherent randomness. The peak Δ*G* value decreased and the device-to-device variability (red) tightened from the second to the sixth pulse. On the other hand, the inherent randomness remained relatively constant. This resulted in the tail of total distribution (blue) extending into the negative Δ*G* regime, which is undesirable (Fig. 5c). The mean and standard deviation of Δ*G* obtained from the experimental data (shown in black circles and error bars) were compared with the root mean square of inherent randomness (*r*) obtained from the GPR-based methodology (shown in red error bars) as a function of pulse number in Fig. 5d. The total standard deviation became comparable with Δ*G* for incremental weight updates. Since the learning accuracy is known to degrade when the ratio of standard deviation to Δ*G* becomes >1^21^, reduction of variability is indispensable. Our analysis revealed that a large portion of total variability is attributed to inherent randomness of individual devices (~67%) for a mature technology based on the 90 nm CMOS baseline. The median SNR value calculated from inherent randomness is ~35% for PCM devices, which is comparable to our ReRAM device switching at a similar *G* level (cf. Fig. 4b). This indicates that variability due to inherent randomness is a common challenge for ReRAM and PCM for neuromorphic computing applications. Innovations in device and material are needed to suppress this component. Our methodology based on GPR enables precise extraction of inherent randomness from individual devices and provides useful guidelines for further improvement.

## Discussion

We established a practical methodology based on GPR to precisely separate signal and noise components from analog NVM elements with non-linear conductance changes. This solves key technical challenges for characterization of artificial synapses of neuromorphic computing system, namely extraction of switching symmetry and SNR. The methodology is agnostic to switching mechanisms and therefore applicable to various types of NVMs. We applied the methodology to HfO_2_-based ReRAM devices and found the tradeoff between switching symmetry and SNR. Using SF as a guideline, substantial improvement in switching symmetry was achieved compared to reported ReRAM devices in literature. By systematic analysis of 1000 GST-based PCM devices, we clearly demonstrated that a large portion of variability in weight update is attributable to inherent randomness from individual devices and this is the key component to be suppressed in order to achieve high classification accuracy.

Finally, the proposed methodology helps neuromorphic system engineers in two ways depending on phases of technology development. In an exploratory phase, our methodology enables extraction of switching symmetry and SNR from individual devices and expedites search for ideal materials. The conventional methodology requires fabrication of many devices with tight device-to-device variability for extraction of SNR, which is difficult to attain in the early stage when exotic material options need to be screened. In a relatively mature technology phase, our methodology helps find the optimum input signals (e.g., duration and amplitude of pulses) that provide the best switching symmetry (linearity) and SNR within the tradeoff for the entire neuromorphic system.

## Methods

### ReRAM device fabrication and test

We fabricated 2-terminal oxide ReRAM with device dimensions of 50 × 50 μm^2^. First, a SiO_2_ underlayer was grown on a 200 mm Si wafer. Then, a 100 nm-thick TiN film was deposited by reactive sputtering as a bottom electrode, followed by deposition of a HfO_2_ layer by atomic layer deposition as a switching layer where a current conducting filament is formed. We varied the thickness of the switching layer (device A: 5 nm, device B: 4 nm) to investigate its impact on switching symmetry. Next, a 20 nm-thick TiN was deposited by reactive sputtering as a top electrode. The device area was defined by photolithography and reactive ion etching of the TiN electrode. To test switching symmetry and SNR of our ReRAM devices, we applied a sequence of weight update (write) pulses with the same voltage amplitude for each polarity. We used high-resolution source measure unit (SMU) to read the device conductance state between the write pulses. We applied a small read voltage of 0.1 V to prevent disturbance in the resistance state. While keeping the read voltage applied across the device, we took multiple read steps with a 16.67 ms integration time until the measured values read at the instrument stabilized (typically within 3–10 repetitive read measurements in the device resistance range of interest). Then, we chose the last measurement as the representative value. We did not detect random telegraph noise with this read sequence. The write pulses had duration of 100 ns (unless otherwise mentioned) and various voltage amplitudes (set pulse: 1.6–1.7 V; reset pulse: −1.8 to −1.9 V) were compared to investigate the impacts on switching symmetry and SNR. In order to separate noises from weight update and those from weight read, we also carried out read-only test, where only read steps were repeated up to 1000 times without weight updates in between. Our linear regression analysis showed that the residual standard error of read-only trace is 2.51 × 10^−7^ S, which is almost one order lower than that of read-after-write trace (1.38–1.57 × 10^−6^ S). Therefore, we attribute a majority of noise components of our ReRAM devices to inherent randomness in weight updates.

### PCM device fabrication and test

The PCM devices were integrated into a chip fabricated in the 90 nm CMOS technology^36^. The phase-change material is doped Ge_2_Sb_2_Te_5_. The bottom electrode has a radius of ~20 nm and was defined using a sub-lithographic key-hole transfer process^37^. The phase-change material is ~100 nm-thick and extends to the top electrode. All experiments in this work were done on an array comprising 1 million devices, which is organized as a matrix of 512 word lines (WLs) and 2048 bit lines (BLs). The selection of one PCM device is done by serially addressing a WL and a BL. A single selected device can be programmed by forcing a current through the BL with a voltage-controlled current source. For reading a PCM cell, the selected BL is biased to a constant voltage of 0.3 V. The resulting read current is integrated by a capacitor, and the resulting voltage is then digitized by an on-chip 8-bit cyclic ADC. The ADCs are calibrated by means of on-chip reference poly-silicon resistors. As for characterization of incremental device *G* change, each device was first initialized to a state that has almost zero conductance. After the initialization, a set pulse of 70 μA was applied followed by conductance read steps. The read step was repeated 50 times to obtain mean *G* values in order to minimize read noise and to focus on characterization of write noise. This sequence was repeated 20 times to obtain *G* values as a function of pulse numbers.

### GPR-based methodology

The goal of GPR is to learn a probability distribution of the output signal, $y$, conditioned on the input signal, $x$, from data $\left\{ {\left( {x^{\left( n \right)},y^{\left( n \right)}} \right){\mathrm{|}}n = 1, \ldots ,N} \right\}$, where $N$ is the number of samples and the superscript $(n)$ denotes the $n$-th sample in the data. The distribution is given by

3 $$p\left( {y{\mathrm{|}}x} \right) = {\cal N}\left( {y|m\left( x \right),s^2\left( x \right)} \right),$$

4 $$m\left( x \right) = {\mathbf{k}}^{\mathrm{T}}\left( {{\mathbf{K}} + {\mathbf{I}}} \right)^{ - 1}{\mathbf{y}}_N,$$

5 $$s^2\left( x \right) = \sigma ^2\left[ {2 - {\mathbf{k}}^{\mathrm{T}}\left( {{\mathbf{K}} + {\mathbf{I}}} \right)^{ - 1}{\mathbf{k}}} \right],$$

where ${\cal N}\left( {y|m\left( x \right),s^2\left( x \right)} \right)$ denotes the Gaussian distribution of $y$ with the mean $m\left( x \right)$ and the variance $s^2\left( x \right)$. Also, $\sigma ^2$ denotes the variance that corresponds to measurement noise, **I** denotes the identity matrix, and ${\mathbf{y}}_N = \left( {y^{\left( 1 \right)}, \ldots ,y^{\left( N \right)}} \right)^{{\mathrm{T}}}$, where the superscript T denotes the matrix transpose.

The key ingredient of GPR is the kernel matrix ${\mathbf{K}}$, which controls the smoothness of the estimated functional curve. We use a non-dimensional kernel ${\mathbf{K}}$ whose $(i,j)$ element is given by

6 $$K\left( {x^{\left( i \right)},x^{\left( j \right)}} \right)\underline{\underline {\,{\mathrm{def}}\,}} {\mathrm{exp}}\left( { - \frac{{|x^{\left( i \right)} - x^{\left( j \right)}|^2}}{{2\sigma _K^2}}} \right).$$

The $n$-th entry of the $N$-dimensional vector ${\mathbf{k}}(x)$ is also given by $K\left( {x,x^{\left( n \right)}} \right)$. The parameters $\sigma _K^2,\sigma ^2$ are learned from the data, as explained later. The idea is to use the predictive mean, $m\left( x \right)$, at the input value (pulse number) $x$, as a noise-free version of the output signal (*G*).

### Determining GPR parameters

The parameter $\sigma ^2$ is determined by maximizing the log marginalized likelihood^26^, which is given by

7 $$E\left( \sigma \right)\,\underline{\underline {{\mathrm{def}}}} \, - \frac{N}{2}\ln \sigma ^2 - \frac{1}{{2\sigma ^2}}{\mathbf{y}}_N^ \top \left( {{\mathbf{K}} + {\mathbf{I}}} \right)^{ - 1}{\mathbf{y}}_N - \frac{1}{2}{\mathrm{ln}}\,{\mathrm{det}}\left( {{\mathbf{K}} + {\mathbf{I}}} \right) + c,$$

in our parameterization, where $c$ denotes an unimportant constant, and det is the matrix determinant. Assuming $\sigma _K$ is given for now and taking the derivative with respect to $\sigma ^{ - 2}$, we have

8 $$\sigma ^2 = \left( {\frac{1}{N}} \right){\mathbf{y}}_N^{\mathrm{T}}\left( {{\mathbf{K}} + {\mathbf{I}}} \right)^{ - 1}{\mathbf{y}}_N.$$

To compute this, we need a value of $\sigma _K$. In theory, we could find it by maximizing $E$ simultaneously with $\sigma .$ This approach, however, involves a complex non-linear optimization procedure and often results in numerical instability in our application.

Here we propose a practical approach that combines the Bayesian marginalized likelihood maximization with the frequentists’ cross-validation approach. Specifically, to determine $\sigma _K$, we maximize the predictive leave-one-out (LOO) likelihood, as defined by

9 $$L\left( {\sigma _K} \right)\,\underline{\underline {{\mathrm{def}}}} \,\mathop {\sum }\limits_{i = 1}^N {\mathrm{ln}}\,{\cal N}\left( {y^{\left( i \right)}|m_{ - i}\left( {x^{\left( i \right)}} \right),s_{ - i}^2\left( {x^{(i)}} \right)} \right),$$

where $m_{ - i}$ and $s_{ - i}^2$ are the predictive mean and variance of GPR (Eqs. (4) and (5)) obtained from the dataset excluding the *i*-th sample. To find the maximizer of $L\left( {\sigma _K} \right)$, we can leverage the fact that the observed variance does not depend heavily on the input across the entire domain. By replacing $s_{ - i}^2$ with a constant, the LOO likelihood criterion is reduced to the task of finding a minimizer of the mean square of the residual (i.e., *r*), which is easily done independently of $\sigma ^2$. In this study, we use the following procedure and criterion to find an appropriate *σ*_*K*_ value from the experimental data. We vary *σ*_*K*_ to cover a wide range and identify an optimum range where the change of *σ*_*K*_ negligibly affects extracted *r* values. This is practically equivalent to maximizing the predictive LOO likelihood. Our criterion is *r* change of <1% for *σ*_*K*_ change of 10% and this is met with a *σ*_*K*_ value of around $3 \times N$ for our dataset (Supplementary Note 6).

### Data availability

The data that support the findings of this study are available from the corresponding author upon request.

## Electronic supplementary material

Supplementary Information
