# Supplementary material for: Signal and noise extraction from analog memory elements for neuromorphic computing
Source: Nat Commun. 2018 May 29;9:2102. doi: 10.1038/s41467-018-04485-1 (PMC5974407; doi:10.1038/s41467-018-04485-1)
Supplement: Supplementary file 1 — Supplementary Information [file 41467_2018_4485_MOESM1_ESM.pdf]

Supplementary Information

Signal and Noise Extraction from Analog Memory Elements for Neuromorphic Computing

Gong et al.

Supplementary Note 1

The distribution of noise in our experimental data for ReRAM (the same device used in Figure 3 of **Characterization of NVM elements** section) was investigated. The histograms of *r* (residual) for the up trace (set) and the down trace (reset) are shown in Figure 1. The fitting with a Gaussian function is shown in blue lines. As one can see, both closely follow Gaussian distributions. This is consistent with one of the assumptions for GPR.

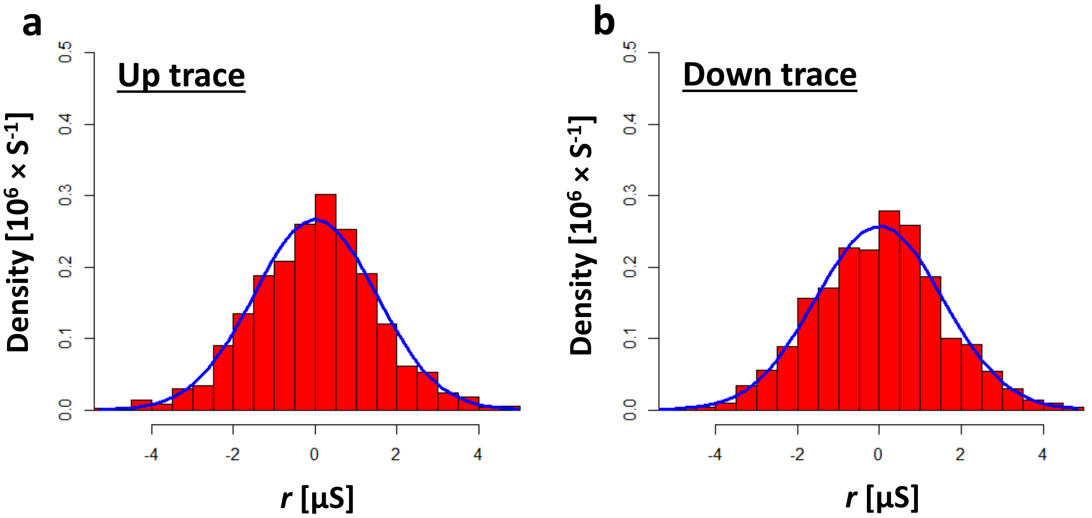

Figure 1 Statistical distribution of *r* values from ReRAM. (a) Histogram of *r* values extracted from the up trace in Figure 3b. (b) Histogram of *r* values extracted from the down trace in Figure 3b. The fitting with a Gaussian function is shown in blue lines.

Supplementary Note 2

The original experimental data for our ReRAM devices consist of 1000 pulse numbers and corresponding *G* values. We divided the original data into training data and verification data with various ratios. For example, if we label 1000 original data from 1 to 1000, each number of 4k (k from 1 to 250) is included in verification data, while remaining data (i.e., 4k-3, 4k-2, 4k-1, k from 1 to 250) are used as training data in the case of ratio of 3 (number of training data over number of verification data). After we chose training data and verification data from the original data, we applied the GPR-based methodology on the training data and the verification data, respectively. As shown in Figure 2, the median *r* values extracted from each data set remained almost constant when we changed the ratio of the number of the training data and the verification data from 1 to 9, which indicates that the methodology extracts the true features irrespective of sampling size.

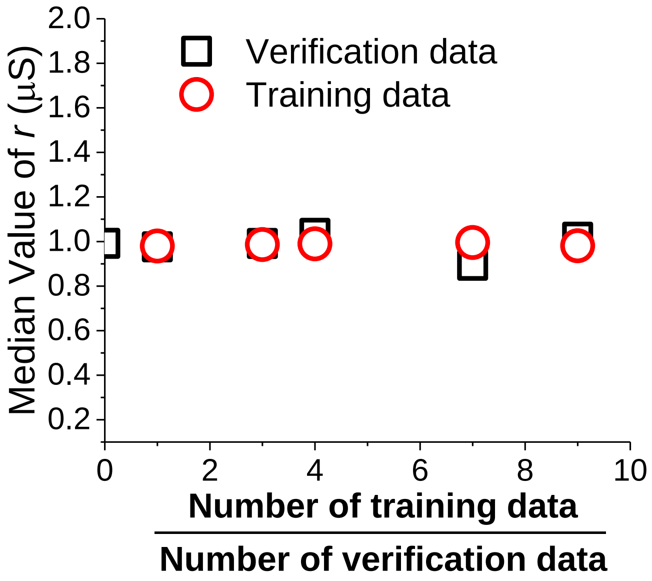

Figure 2 Cross-validation of GPR based methodology. Median *r* value as a function of ratio of number of training data and verification data.

Supplementary Note 3

We used input pulses with duration of 100 ns in the main experiment for our ReRAM devices (see also Supplementary Note 6). We additionally tested input pulses with duration of 5, 20, and 50 ns on ReRAM Device B. We applied 200 consecutive set pulses (+1.0 V), followed by 200 consecutive reset pulses (-1.4 V) for each pulse duration. Please note that we used a cross-finger device structure with the device area of 5×5 μm^2 to enable fast pulse measurement, therefore the voltage amplitudes used in this experiment are slightly different from those for the main experiment with 100 ns pulses.

We varied the σ_k value in the range between 200 and 1400 at intervals of 30 to find the optimum value for GPR fitting. Please refer to Methods for the objective of this procedure. The *r versus* σ_K plot for each pulse duration is shown in Figure 3.1. We found local minima with sufficiently wide flat regions around σ_K of 400-600 for all pulse conditions. σ_K values below the flat regions provided smaller *r* values due to over-fitting, so we avoided them. The experimental *G versus* pulse number plots with predicted noise-free signals (red lines) are shown in Figure 3.2. We used σ_K of 600 for all pulse conditions.

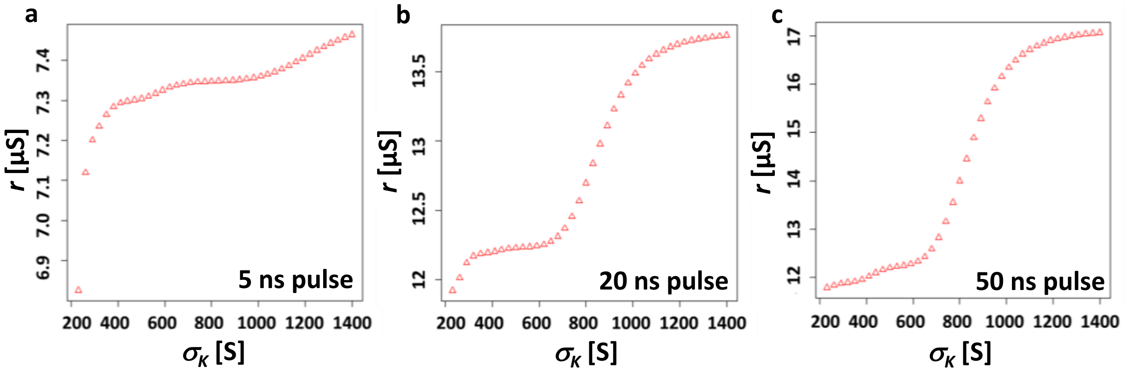

Figure 3.1 Stability of extracted *r* values against varying σ_K values for ReRAM with different pulse durations. Median *r* value as a function of σ_K for the GPR-fitting with pulse durations of (a) 5 ns (b) 20 ns (c) 50 ns.

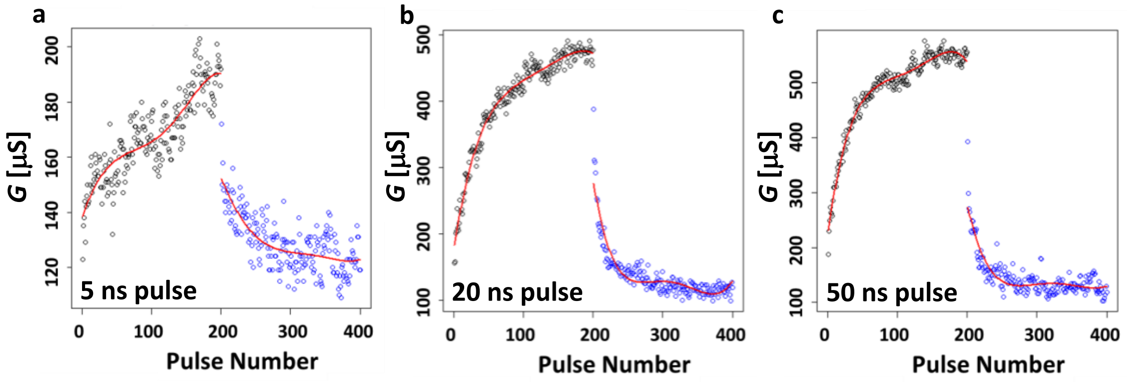

Figure 3.2 GPR fitting for different pulse durations. Device *G* as a function of pulse number for set (black) and reset (blue) pulse sequences with pulse durations of (a) 5 ns (b) 20 ns (c) 50 ns. The predicted noise-free signals are shown in red lines.

As shown above, we demonstrated a robustness of our GPR-based methodology for the variation of pulse duration by a factor of 20 and down to 5 ns, covering the range of interest for neuromorphic computing. We think that this is because the basic assumptions of GPR (i.e. smoothness of functional curve, Gaussian distribution of noise) are not violated with the change of pulse duration as far as the device operation remains in the analog switching domain.

Supplementary Note 4

We tested ReRAM Device B at 85°C to check the stability of our fitting methodology against the change in measurement temperature. We applied 200 consecutive set pulses (+1.2 V), followed by 200 consecutive reset pulses (-1.2 V) with duration of 100 ns. Please refer to Supplementary Note 3 for the trends at room temperature for the same sample. We varied the σ_K value in the range between 200 and 1400 at intervals of 30 to find the optimum value for GPR fitting. The *r versus* σ_K plot is shown in Figure 4.1.

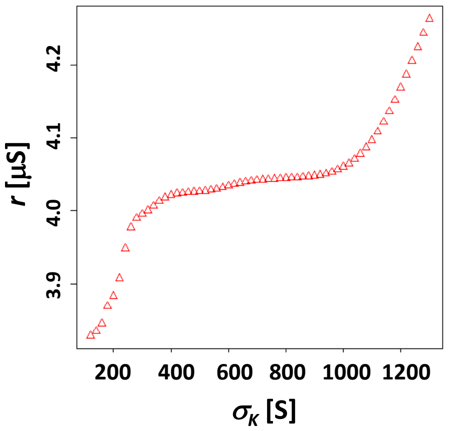

Figure 4.1 Stability of extracted *r* values against varying σ_K values for ReRAM tested at 85°C. Median *r* value as a function of σ_K for the GPR-fitting for device type B (HfO_2 thickness 4 nm). 200 consecutive set pulses (+1.2 V), followed by 200 consecutive reset pulses (-1.2 V), were applied.

We found a local minimum with a sufficiently wide flat region around σ_K of 400-1000. The experimental *G versus* pulse number plot with predicted noise-free signals (red lines) are shown in Figure 4.2. We used σ_K of 600 for this fitting. The optimum σ_K value is around 3 × *N* (pulse number) for the measurement at 85°C, which is consistent with the trend at room temperature (Supplementary Note 6).

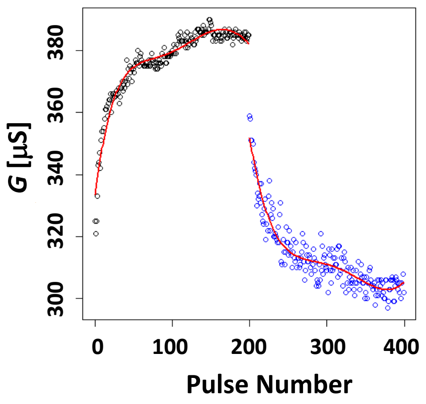

Figure 4.2 GPR fitting for ReRAM tested at 85°C. Device *G* as a function of pulse number for set (black) and reset (blue) sequences. The predicted noise-free signals are shown in red lines.

This indicates that the assumptions of GPR fitting are not violated at practical test temperatures and our fitting is robust against the change in temperature.

Supplementary Note 5

We checked the reproducibility of switching symmetry up to 10 different devices of device type B (HfO_2 thickness 4 nm) to show the validity of our discussion on the trend. For this test, 200 consecutive set pulses (+1.7 V), followed by 200 consecutive reset pulses (-1.9 V), were applied and *SF* values were extracted for each device. The cumulative distribution function (CDF) of |SF| for 10 different devices are shown in Figure 5.

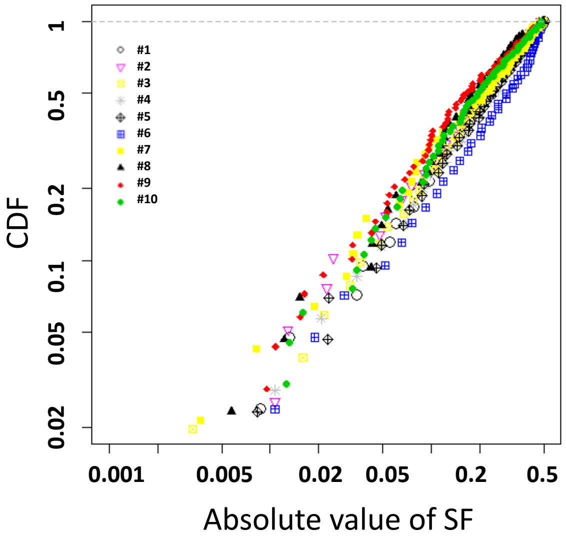

Figure 5 Reproducibility of switching symmetry. The cumulative distribution function (CDF) of |*SF*| for 10 different devices obtained for device type B (HfO_2 thickness 4 nm). 200 consecutive set pulses (+1.7 V), followed by 200 consecutive reset pulses (-1.9 V), were applied.

We confirmed good reproducibility of the trend. When we varied the pulse conditions, we applied them on the same device and extracted SNR and SF values for each condition to exclude device-to-device variability. For comparison of different device conditions, we varied the metal oxide thickness in a wide range (Device A: 5 nm, Device B: 4nm) to make the difference large enough compared to device-to-device variability. The improvement for Device A in Figure 4c is larger than the variability shown above. Based on this, we think that our conclusions on the device trends for ReRAM are valid.

Supplementary Note 6

The experimental data for our ReRAM devices (pulse duration of 100 ns) consist of 1000 pulse numbers (N) and corresponding G values. We performed the GPR-fitting using σ_K values ranging from 1000 to 5000 at intervals of 100. Then, we extracted a median r value across the entire domain for each fitting and plotted as a function of σ_K. As one can see in Figure 6.1, the median r values showed a flat trend around a σ_K value of 3000 ($3 \times N$). In this flat region, the change in median r value is < 1% for σ_K change of 10%, indicating that the GPR-fitting is stable and the inherent feature is extracted. We used this criterion for optimization of σ_K for all data sets including literature data.

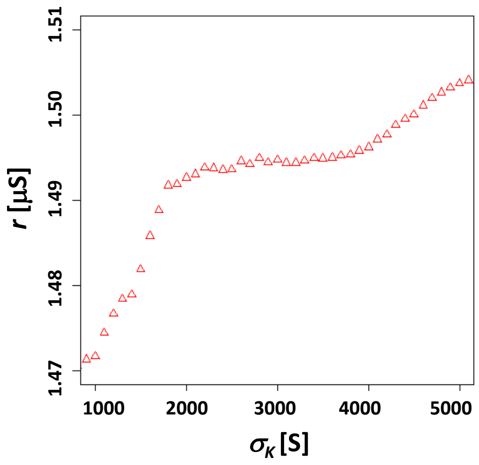

Figure 6.1 Stability of extracted *r* values against varying σ_K values for ReRAM. Median *r* value as a function of σ_K for the GPR-fitting.

For the analysis of PCM devices, we varied the σ_K value in the range between 20 and 140 at intervals of 3 to find the optimum value for GPR fitting. The *r versus* σ_K plot for one of the thousand PCM devices is shown in Figure 6.2. The plateau of r values in a wide range of σ_K values was confirmed. For consistency with the analysis of ReRAM devices, we used $\sigma_K = 3 \times N$ (i.e. σ_K of 60) for statistical analysis of PCM data.

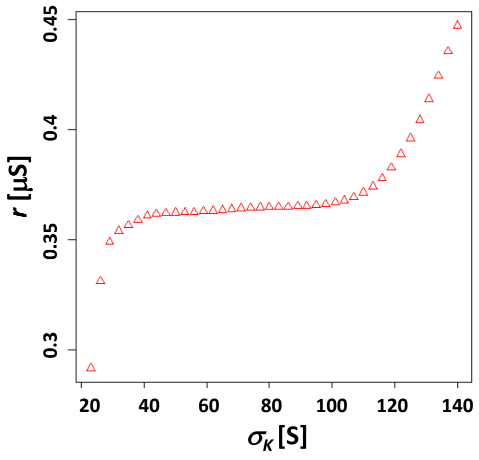

Figure 6.2 Stability of extracted *r* values with varying σ_K values for PCM. Median *r* value as a function of σ_K for the GPR-fitting.
